# OFDM Network Optimization Using a QPSK Based on a Wind-Driven Genetic Algorithm

**DOI:** 10.3390/s22166174

**Published:** 2022-08-18

**Authors:** Yanxia Sun, Chikomborero Shambare, OdunAyo Imoru

**Affiliations:** 1 Department of Electrical and Electronic Engineering Science, University of Johannesburg, Johannesburg 2006, South Africa; 2 Department of Electrical and Computer Engineering, University of Namibia (JEDS Campus), Ongwediva 15006, Namibia

**Keywords:** bit error rate, carrier offset drift, genetic algorithm, orthogonal frequency division multiplexing, phase ambiguity, quadrature phase-shift keying, wind-driven optimization

## Abstract

Quadrature phase shift keying (QPSK) is a digital modulation technique that transmits data at a constant frequency whilst varying the phases of the carrier signal. QPSK is one of the fundamental modulation schemes for orthogonal frequency division multiplexing systems (OFDM). It is a stable modulation technique with good spectral efficiency. However, during transmission, the carrier signal can undergo numerous phase changes. This creates phase ambiguity problems at the receiver end. This results in inter-symbol interference (ISI) and a high bit error rate (BER). In this paper, the wind-driven optimization was incorporated into the genetic algorithm (GA) as its population selection function. This hybrid algorithm was used to determine the phase assignments for the QPSK. The developed QPSK was implemented on an OFDM network and the message signal was recovered at more than 92% accuracy in a noisy Rayleigh fading channel and 100% accuracy in a noiseless channel. The enhancements greatly mitigated phase ambiguity and bit errors.

## 1. Introduction

The continuous advancements in wireless communication technology increase the need for efficient and dependable communication networks [1]. The networks need to have more data capacity and low bit errors at the receiver end [2]. Orthogonal frequency division multiplexing (OFDM) is a digital data transmission technique that encodes data onto numerous carrier frequencies for transmission over a common channel [3]. OFDM is used in applications such as wireless networks, digital television broadcasting, power line communication networks, and 4th and 5th generation mobile communications [4]. A communication signal has three basic characteristics that can be manipulated, i.e., phase, frequency, and amplitude. These can be varied depending on the transmission modulation scheme being used in the communication system [5].

Quadrature phase-shift keying (QPSK) is a phase-altering modulation scheme used for modulating OFDM subcarriers. QPSK uses four phases with each phase being assigned a unique number of binary digits [6]. To ensure good data recovery, the demodulator is custom designed for the symbol sets used during modulation. Regardless of its popularity, the QPSK technique greatly suffers from phase ambiguity and carrier offset drift [7]. This automatically introduces inter-symbol interference (ISI) and inter-carrier interference (ICI) on the transmitted signal [8]. However, to further improve on the progress made in developing the QPSK technique, other enhancement methods need to be utilized [9]. 

The genetic algorithm (GA) and the wind-driven optimization (WDO) are metaheuristic tools that give precise solutions to complex optimization and mathematical problems [10]. In their problem evaluation procedures, both algorithms begin with a multitude of solutions and use the Pareto sense in prioritizing the solutions [11].

In this paper, the two algorithms were merged into a hybrid algorithm. The WDO was incorporated into the GA as its selection function. The hybrid genetic algorithm wind-driven optimization (GAWDO) was used in an analytical modeling technique for deducing and designating the optimum phases for a QPSK signal in an OFDM network. When compared to the conventional QPSK, the GAWDO-assisted QPSK showed better results in both noisy and noiseless OFDM networks. The states and operating conditions that were beyond the scope of the developed QPSK could also be noted.

The rest of the paper is organized as follows: Section 2 gives the conducted literature survey which covers related work and the emanating research questions. Section 3 gives a detailed description of the proposed GAWDO for OFDM network optimization and the detailed QPSK enhancements. Section 4 presents the performance evaluation and Section 5 gives the results and their analysis. Finally, Section 6 gives the conclusion of the paper.

## 2. Materials and Methods

### 2.1. Related Work

This section discusses some previous works that studied QPSK modulation, OFDM channel performance improvement, and bandwidth optimization in fading channels. An OFDM signal is obtained by performing an Inverse Fast Fourier Transform (IFFT) on a QPSK signal followed by the addition of a cyclic prefix (CP) [12]. OFDM modulation is either conducted using quadrature phase-shift keying (QPSK) or quadrature amplitude modulation (4-QAM) [13]. These two modulation techniques can be modified into higher-order modulation schemes, e.g., QPSK constellations can be doubled resulting in 8-PSK and 4-QAM can also be increased up to 64-QAM before major distortions start to emerge. In [14], a hybrid extended phase shift modulation strategy is introduced to bring stability to a system that is dominated by non-linear components. This methodology shows that the conventional modulation techniques can be enhanced resulting in better channel performance. The authors successfully had channel performance improvement by enhancing the modulation techniques, but their main challenge was with optimal parameter selection. The authors in [15] presented a methodology of having a constant envelope waveform output from modulation that would be fed into the channel. The methodology offered good spectral efficiency. However, since most data are received in digital form as binary digits, this methodology did not fully address the mitigation of bit errors in communication systems. The authors in [16] presented a simulation methodology for OFDM networks. The methodology was intended to break down an OFDM system and highlight the blocks from where distortions or errors might emanate. The methodology proved to be very useful for OFDM network analysis but did not offer a substantial number of solutions to the problems encountered on the various channel sub-blocks, e.g., noise and attenuation. The authors in [17] did a performance analysis for PSK and ASK modulation under uncertain and noisy conditions. The analysis showed that the customary PSK has better output than other modulation techniques. However, regardless of its displayed superiority, it still had massive bit errors and distortions resulting in loss of information. This raised more questions on whether the customary PSK should be considered for 5G and the subsequent technologies considering the need for high data throughput and bandwidth efficiency. The authors challenged fellow researchers to come up with further PSK enhancement techniques. In [18], the authors did a raw comparison of PSK and QAM in an OFDM system. They compared these modulation techniques in their customary form, i.e., without any enhancements. The QPSK technique gave stable results, but it was not sturdy against ICI and ISI. Phase ambiguity was its biggest drawback. The amplitude modulation techniques produced comparably good results, but they were not adaptive to channel conditions and were also very susceptible to synchronization errors. The authors in [19,20] analyzed the various encoding techniques and challenged fellow researchers to develop or enhance the customary techniques. They recommended that fellow researchers should start by enhancing the basic modulation techniques, i.e., QPSK or 4-QAM before attempting the higher-order modulation techniques so that any synchronization errors can be easily noted and rectified. In [21], a hybrid QPSK modulation technique was presented. This technique is made by rearranging the phases and amplitudes of the modulating signal. This was done in an attempt to mitigate detection errors in fading channels. This technique gave significantly better results in noisy optical channels than the conventional modulation techniques. Nonetheless, the technique suffered greatly from phase ambiguity resulting in some errors.

Regardless of the weaknesses in the various modulation enhancement techniques, the authors in the literature reviewed above give useful insights on where to start and how to manipulate the conventional modulation techniques if one wants to significantly improve them and obtain better OFDM channel data throughput.

### 2.2. Research Questions

Based on the literature reviewed in Section 2, the quest to improve error performance, increase data throughput, raise bandwidth efficiency, step up channel capacity, and elevate OFDM network reliability creates a need to address the following questions [6,8,9,10,11,14,15,19]:

- How can the WDO be successfully incorporated into the GA to help mitigate the weaknesses of the GA, e.g., selecting the most elite parents for subsequent populations thus mitigating premature convergence and promoting diverse exploration [10,11]?
- How can the developed hybrid GAWDO be used to deduce and assign optimum phase values for QPSK in an attempt to mitigate QPSK phase ambiguity [6,17,18]?
- How can the QPSK based on the GAWDO be further modified to eliminate carrier offset drift?
- How can OFDM channel efficiency be increased by optimizing a QPSK signal [8,9]?

## 3. GAWDO for OFDM Network Optimization

### 3.1. The Proposed GAWDO

The GA and the WDO are optimization techniques that are based on the principles of evolution. They commence with a set of solutions that undergo initialization, selection, mutation, and recombination until the most elite and rational settlement has been attained [22]. This makes them superior to conventional methods. The WDO is a straightforward and fast converging algorithm [23]. However, the GA struggles with the selection of elite individuals which often leads to stalling and untimely convergence [24,25].

In this paper, a hybrid genetic algorithm wind-driven optimization (GAWDO) technique is introduced. The main aim of hybridization is to lessen the limitations of an algorithm with diversification aspects of another algorithm. This hybrid algorithm was designed to give better quality results than those produced by the traditional GA and WDO. The GAWDO was created based on incorporating the WDO as part of the GA selection function for the following reasons:

- To continuously influence the number and quality of individuals that can be created and given the responsibility of reproduction at every evolution stage. This would reduce the loss of good genetic material. Since the GA is a multipath search algorithm, this would reduce the chances of trapping in suboptimal minima [26,27].
- To select and maintain a healthy population which would ensure search efficiency until termination of the optimization processes. This would also help the algorithm to effectively explore all the search spaces where the probability of finding optimum solutions is highest [10,24,28].
- To select and maintain parents with the best structural variations. This implies more diversity since the probability of replacing a gene with a similar/redundant structure will be small. This would ensure maximum population diversity [12,17,18].
- To select parents based on their expectations. The best parents with high expectation values would be given more reproduction chances in descending order. This would mitigate stalling which greatly exists in the conventional GA when scores or expectation values are similar because they are chosen using probabilities [10,26,29].
- To ensure good survival rates of the best individuals. Having the best individuals would give more assurance that all the desired search space would be fully exploited [30,31].

**Figure 1:**
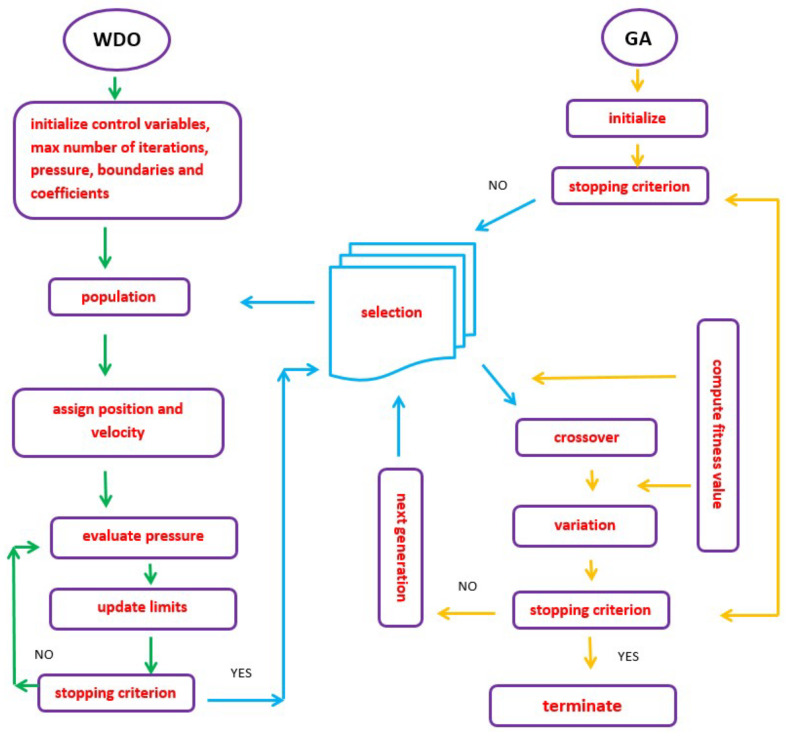
GAWDO structure.

The hybrid algorithm was developed by merging the two algorithms as per Figure 1.

### 3.2. Quadrature Phase-Shift Keying Enhancement

QPSK is a digital modulation scheme that transmits data by varying the phase of the carrier signal. The carrier wave variations have to be with great precision [6]. QPSK can also be referred to as 4-QAM (quadrature amplitude modulation). The root concepts of QPSK and 4-QAM are different, but the resulting modulated waves are the same [19,20]. QPSK uses four points on a constellation diagram. It can encode 2 bits per symbol. QPSK systems remain susceptible to noise and high bit errors because they use binary data [18]. For QPSK systems to give optimum results, linearity needs to be maintained in the system. However, this is impossible to achieve due to the presence of components that present transient and non-linear behavior, e.g., high-power amplifiers [21]. QPSK also encounters phase ambiguity and carrier offset drift. This will automatically introduce ISI and ICI to the transmitted signal [8]. Considering that all channels already consist of noise, these interferences further increase the signal distortion and hinder demodulation of the transmitted signal with minimum bit errors. However, QPSK is highly preferred for OFDM systems because it ensures spectral efficiency, good noise immunity, and a low probability of error as compared to other modulation techniques [32]. These advantages that it presents are very important because communication channels can only use a limited amount of bandwidth issued by the Federal Communications Commission [33].

In OFDM systems, the transmitted QPSK is defined by the following benchmark functions [4,8,19] in Equations (1)–(4):

(1) $$S_{i}\left(t\right)=\sqrt{\frac{2E}{T}}\left[2\pi f_{c}t+\left(2i-1\right)\frac{\pi }{4}\right]$$

for 0≤t<T & $f_{c}=\frac{n_{c}}{T_{b}}$ whereby $f_{c}$ is the carrier frequency, $n_{c}$ is the number of carriers, and *T* is the time period, the pair of quadrature carriers is defined using the following benchmarks:

(2) $${ɸ}_{1}\left(t\right)=\sqrt{\frac{2}{T}}\;\cos\left(2\pi f_{c}t\;\right)$$

(3) $${ɸ}_{2}\left(t\right)=\sqrt{\frac{2}{T}}\;\sin\left(2\pi f_{c}t\;\right)$$

for 0≤t<T & $f_{c}=\frac{n_{c}}{T}$

The bit error probability of QPSK over an additive white Gaussian noise (AWGN) channel is given by the following benchmark functions:

(4) $$P_{b}=\frac{1}{2}erfc\;\left(\sqrt{\frac{E_{b}}{N_{0}}}\;\;\right)$$

whereby $\frac{E_{b}}{N_{0}}$ is the energy per bit to noise spectral density.

**Figure 2:**
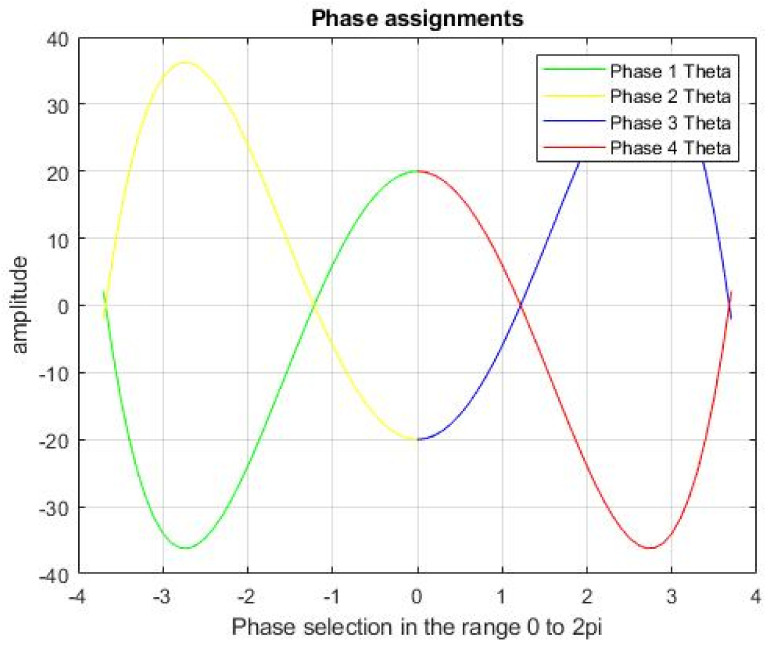
QPSK phase assignment domains.

In this research, the GAWDO was used for deducing and assigning the phase components of the QPSK. The phase assignments can be values within the range of 0 to 2π. In this research, a quartic function was used to determine the values that would be considered by the GAWDO. The quartic function was reflected along the X and Y axis so that it would ensure the same domain of values but in four different constellations as shown in Figure 2. During transmission, the conventional QPSK uses four fixed points on a constellation diagram which greatly differs from the proposed methodology which uses varying but optimum points.

The graph shows that the GAWDO values would mostly be in the range of 0 to 4 and would not necessarily extend from 0 up to the maximum which is 2π. Values beyond 4 would cause phase ambiguity within the system. The quartic function is given in Equation (5).

(5) $$f\left(x\right)=x^{4}-15x^{2}+20$$

The radian value ‘Φ’ obtained by the GAWDO would be incorporated into the QPSK phase ‘*ϴ*’ from Equations (1)–(3) as shown below in Equations (6) and (7):

(6) $$ϴ=\left[\frac{2\times \pi \times ᾱ}{\lambda }\;\right]+ɸ$$

(7) $$\beta =e^{i\;\left(ϴ+{ϴ}_{int}\right)}$$

This is where ᾱ is the message signal and *λ* is the alphabet size. *λ* is an integer power of 2. The message signal ᾱ would consist of integers between 0 and *λ* − 1. For two or more dimensional signals, each column would be considered a channel. ${ϴ}_{int}$ is the initial phase. *β* will be the final output, i.e., the complex envelope of the modulation of the message signal.

During demodulation, the received signal is de-rotated as expressed in Equation (8). The de-rotation would be done using element-wise array multiplication since the demodulator receives parallel data streams. The radian value ‘Φ’ is subtracted and if need be, the signal is converted from radians to degrees.

(8) $$\beta =\beta \;.\times e^{i\;\left(ϴ+{ϴ}_{int}\right)}$$

**Figure 3:**
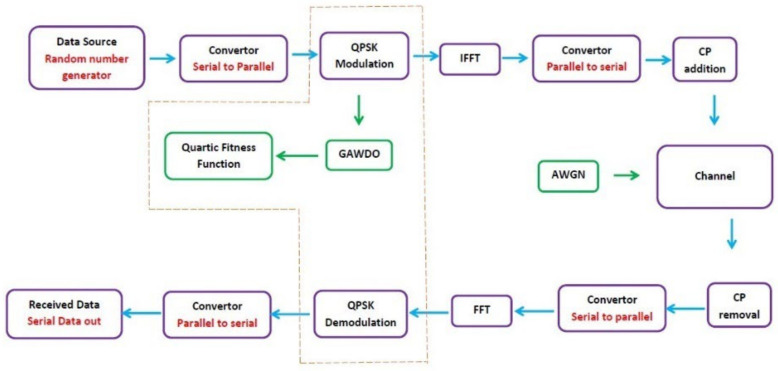
OFDM network sub-blocks and flow chart.

The OFDM network that is optimized has the sub-blocks highlighted in the flow chart in Figure 3. The incorporation of noise and the developed GAWDO into the system is clearly articulated [3,4,12,20].

## 4. Performance Evaluation

### 4.1. Simulation Settings

All the algorithms were executed within MATLAB R2021a using an Intel(R) Core (TM) i7-8665U (2.2 GHz) CPU with 16GB RAM installed on Microsoft Windows 10. The parameters of the wind-driven genetic algorithm are given in Table 1.

### 4.2. Testing of the Algorithms

Before incorporating the GAWDO into the QPSK, it was first tested on the Rastrigin and Ackley benchmark functions given in Equations (9) and (10), respectively.

(9) $$g\left(x\right)=20+X_{1}{}^{2}+X_{2}{}^{2}-10\left(\cos2{\pi X}_{1}+\cos2{\pi X}_{2}\right)$$

(10) $$g\left(x,y\right)=-20\exp[-0.2\sqrt{0.5\left(x^{2}+y^{2}\right)}-\exp\left[0.5\left(\cos2\pi x+\cos2\pi y\right)\right]+\exp\left(1\right)+20$$

The GAWDO was tested alongside the conventional WDO and GA for comparison. The conventional WDO and GA were derived from [23,26,34,35]. These preliminary tests were done to confirm the robustness and accuracy of the various algorithms. All the algorithms were run five times and their results are presented in Table 2 and Table 3.

Both benchmark functions given above have a global minimum of [0;0]. From Table 2, it can be seen that the GA and the WDO often struggle with retaining the global minima. They tend to converge in local minima. In optimization cases, an algorithm that converges poorly and settles to local minima is regarded as inaccurate and unreliable. That particular algorithm must not be given much priority with regards to optimizing much more sophisticated problems [11]. Henceforth, the GAWDO was the only algorithm that was further used on the QPSK.

### 4.3. Simulation Settings

The model of the network used in this work was created based on the illustration in Figure 3 and channel properties in [2,4,13,36,37]. The model resembles a basic communication network. It has all the basic blocks to fulfil all the requirements of an OFDM network. At the source, the QPSK signal constellation is 4. The number of data points varied from 64 to 1024. The OFDM block size is varied accordingly from 4 to 32. The CP length is a rounded value of 10% of the block size and the number of Fourier points is equal to the block size. The equations used at each stage considered ‘*N*’ parallel streams of data points with $X\left(k\right)$ being the transmitted symbol by carrier ‘*k*’ for k=0,1,2,3,…N−1. The OFDM signal ‘S’ for the symbols ‘*s*’ that are assigned to a chosen constellation at a given frequency is given by (11):

(11) $$S=\overset{N-1}{\underset{n=0}{\sum }}\;[\;s\times \sin\left(2\pi ft\right)\;]$$

The Fourier transform breaks down the modulated signal into different frequency packets by variably multiplying the signal with well-defined sinusoids. This converts the signal from time to frequency domain. The IFFT highlighted in Figure 3 performs the conversion process on the QPSK modulated signal as expressed in Equation (12):

(12) $$x\left(n\right)=\{\overset{N-1}{\underset{n=0}{\sum }}X\left(k\right)\sin\left(2\pi k_{n}\right)-j\overset{N-1}{\underset{n=0}{\sum }}X\left(k\right)\cos\left(2\pi k_{n}\right)\}$$

To convert back to the original message signal, an FFT is performed. It differs from the IFFT on the coefficient and addition sign. It reverses the IFFT. The FFT which does the signal conversion on the channel output signal has the form given in Equation (13):

(13) $$X\left(k\right)=\{\overset{N-1}{\underset{n=0}{\sum }}x\left(n\right)\sin\left(2\pi k_{n}\right)+\;j\overset{N-1}{\underset{n=0}{\sum }}x\left(n\right)\cos\left(2\pi k_{n}\right)\}$$

Since most signals are in the time domain, the IFFT is used at the transmission stage and FFT at the receiver. This is interchangeable depending on the domain of the received signal. The corresponding dual will have to be used at the receiving end. The Rayleigh fading channel used in this research is based on Jake’s model whereby the fading of the symbol ‘*k*’ over time ‘*t*’ is given in Equation (14):

(14) $$Y\left(t,k\right)=2\sqrt{2\;}\left[\overset{M}{\underset{n=1}{\sum }}\left(\rho \right)\left(\delta \right)+\frac{1}{\sqrt{2}}\;\left(\tau \right)\left(\varphi \right)\;\right]$$

where ρ is $\left(\cos\beta_{n}+\;\mathrm{jsin}\beta_{n}\right)$, δ is $\left(\cos2\pi f_{n}t+\lambda_{n,k}\right)$, τ is $\left(\cos\alpha +\mathrm{jsin}\alpha \right)$, and φ is $\left(\cos2\pi f_{d}t\right)$. In the simulations, α was mostly set to 0 and $\beta_{n}$ was carefully selected to avoid cross-correlation between the real and imaginary parts of $Y\left(t\right)$. $\lambda_{n}$ was also set at 0 because the simulations were executed in a single-path channel.

## 5. Results

### 5.1. Noiseless Channel Simulation Outputs

The simulation charts below are for a noiseless OFDM network without any fading characteristics or AWGN.

**Figure 4:**
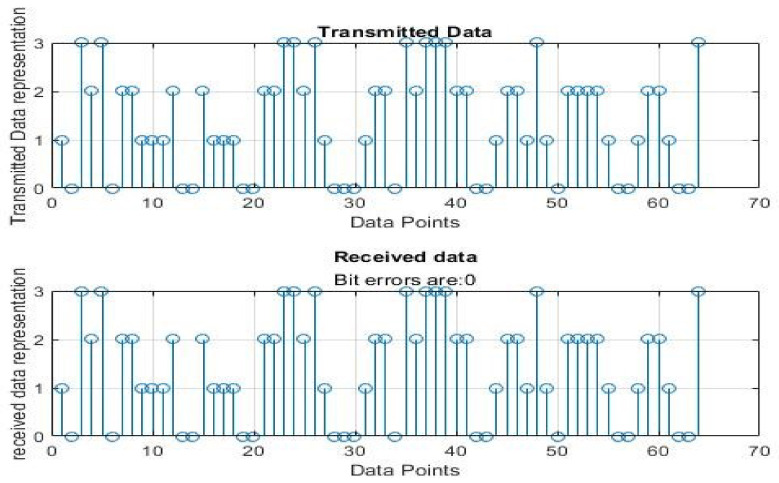
GAWDO QPSK output for 64 data points in a noiseless channel.

**Figure 5:**
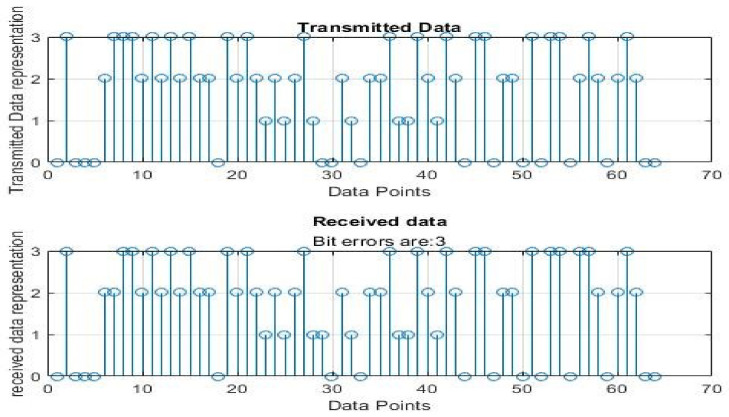
Customary QPSK output for 64 data points in a noiseless channel.

**Figure 6:**
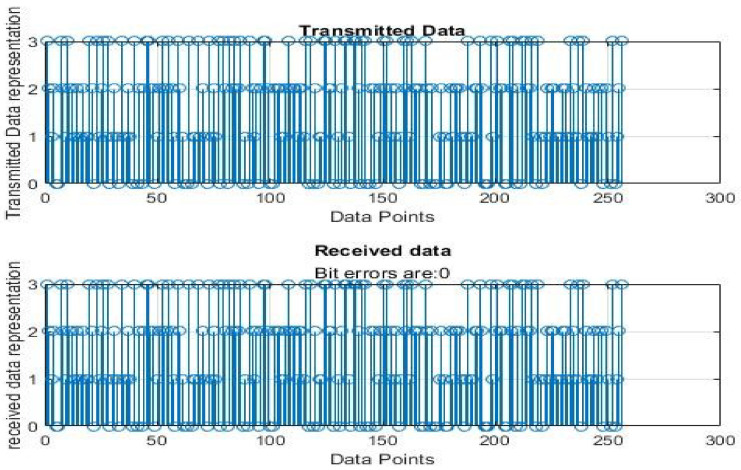
GAWDO QPSK output for 256 data points in a noiseless channel.

**Figure 7:**
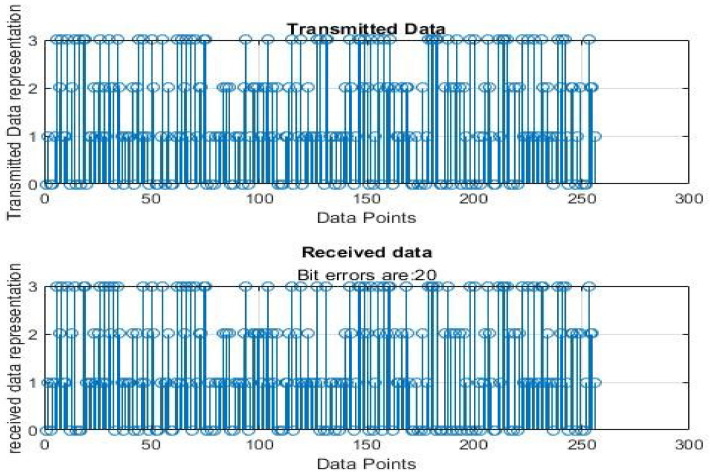
Customary QPSK output for 256 data points in a noiseless channel.

**Figure 8:**
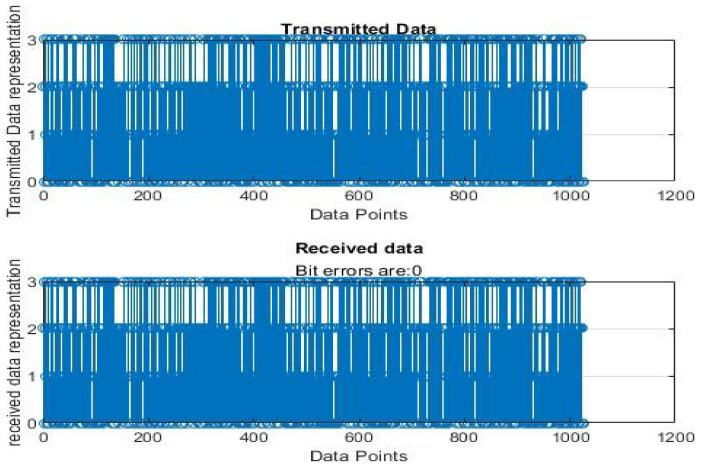
GAWDO QPSK output for 1024 data points in a noiseless channel.

**Figure 9:**
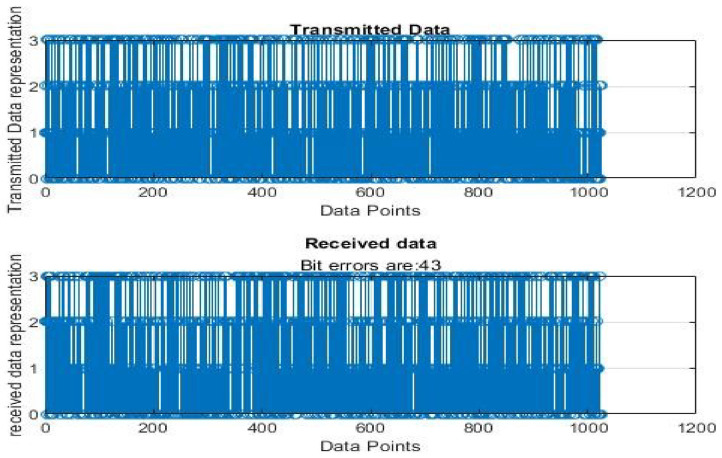
Customary QPSK output for 1024 data points in a noiseless channel.

Figure 4, Figure 5, Figure 6, Figure 7, Figure 8 and Figure 9 show the output of running the two different QPSKs in a noiseless channel. In the noiseless channel, the transmitted signal is what is expected at the receiver stage after demodulation. From the figures above, the GAWDO QPSK encounters no bit errors whereas the customary QPSK has bit errors regardless of how many data points have been transmitted.

This reflects weakness within the system itself and its data transmission processes since no noise or external interferences existed in the network. The bit errors increase with an increase in the number of transmitted data points which means that the conventional QPSK is not very reliable for multi-user systems which need a high data throughput.

### 5.2. Noisy Channel Simulation Outputs

The simulation charts below are for a noisy channel with additive white Gaussian noise (AWGN) and Rayleigh fading characteristics.

**Figure 10:**
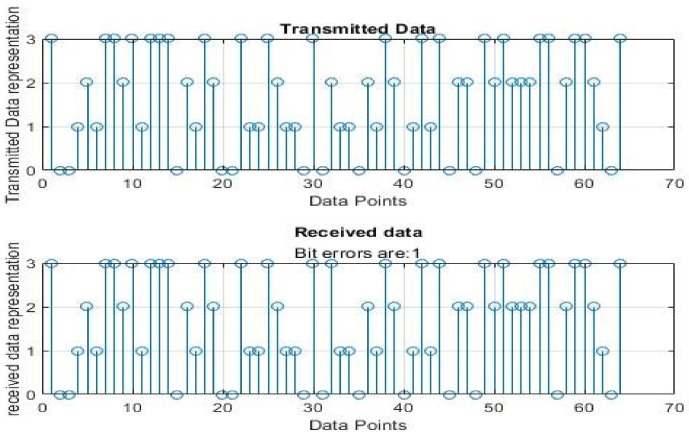
GAWDO QPSK output for 64 data points in a noisy channel.

**Figure 11:**
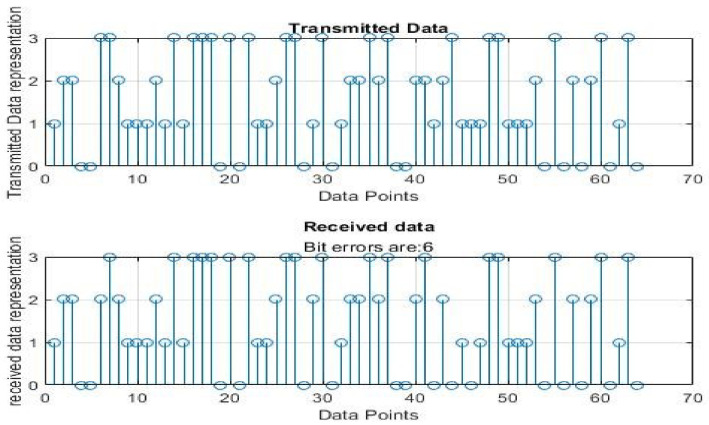
Customary QPSK output for 64 data points in a noisy channel.

**Figure 12:**
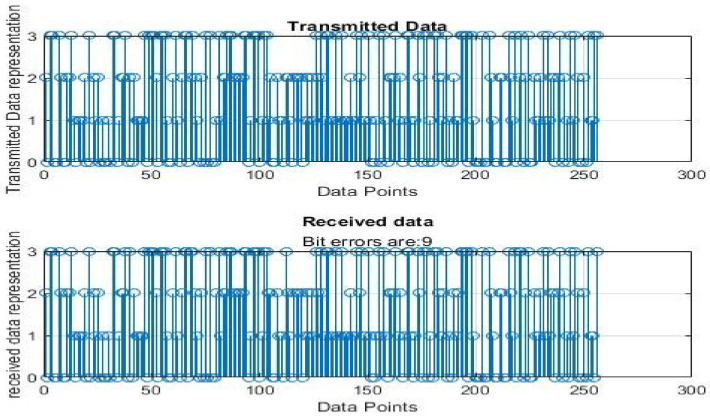
GAWDO QPSK output for 256 data points in a noisy channel.

**Figure 13:**
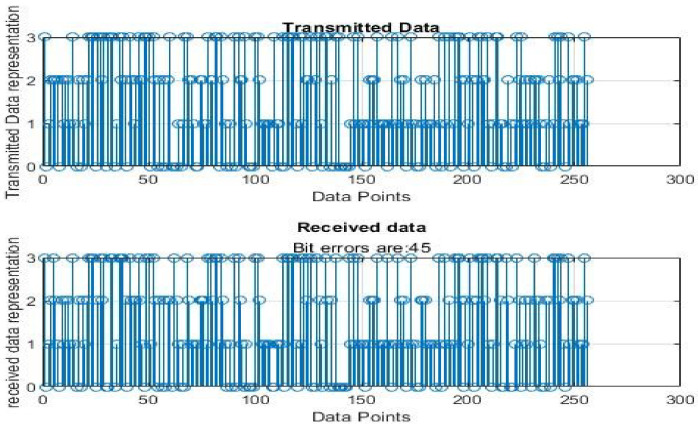
Customary QPSK output for 256 data points in a noisy channel.

**Figure 14:**
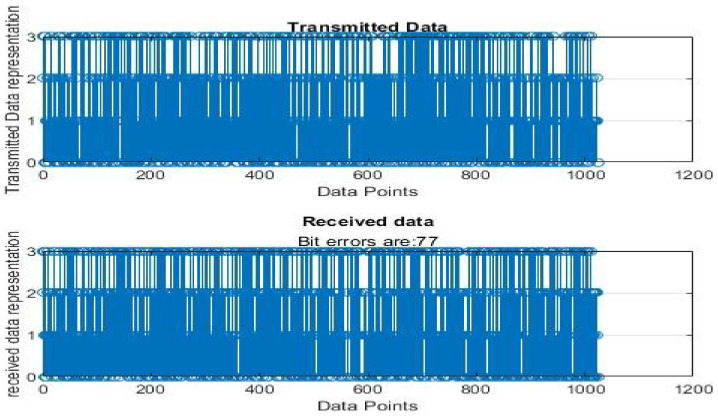
GAWDO QPSK output for 1024 data points in a noisy channel.

**Figure 15:**
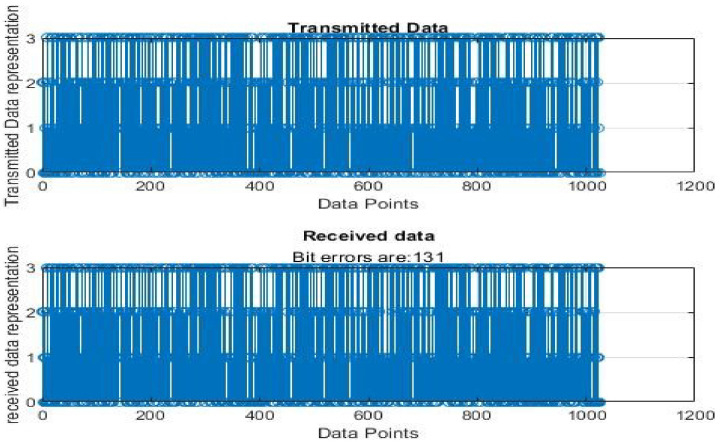
Customary QPSK output for 1024 data points in a noisy channel.

Figure 10, Figure 11, Figure 12, Figure 13, Figure 14 and Figure 15 show the output of simulating the two different QPSKs in a Rayleigh fading channel with AWGN. Distortions are expected in a fading channel [13,37]. The severity of the distortions is determined by the weaknesses or robustness of the system. According to Equation (4) in Section 3.2, there is an expected bit error probability which is computed based on the energy per bit to noise spectral density ratio. In a system with efficient modulation and demodulation, using an envelope or coherent detector will manage to give an output waveform similar to the input waveform [19,20]. The decision-making module can estimate the transmitted signal. However, if the modulating wave (carrier signal) is not consistent with the expected signal at the demodulation stage, more errors will be present on the received signal. This is the case with the customary QPSK which is showing a high BER in Figure 10, Figure 11, Figure 12, Figure 13, Figure 14 and Figure 15.

### 5.3. Results Analysis

Table 4 and Table 5 give a much clearer comparison of the BER for the two modulation schemes. The channel properties and simulation settings that were used are given in Section 4.

From Table 4 and Table 5, in a noiseless channel, the GAWDO QPSK has no BER. The transmitted signal is recovered with high fidelity. No distortion occurs. For the customary QPSK, it experiences bit errors of up to 7.8%. In a noisy channel, the GAWDO QPSK had bit errors not exceeding 8% but the customary QPSK had bit errors going beyond 17%.

This highlights the challenges that the customary QPSK fails to overcome. These high bit errors are caused by the system failing to maintain linearity even under a noiseless/clean operating scenario. This also shows that carrier offset drift and phase ambiguities remain a major cause for concern since the customary QPSK phases are fixed according to the constellation diagram [7,8]. In a noisy channel, ISI and ICI are rife and this caused the BER to compound significantly. In a real-world scenario where multipath fading is of great detrimental effects, the BER values obtained through simulation by this research can double [21]. This means that as much as the customary QPSK is popular, it does not offer very satisfactory results. From Table 5, in a noisy channel, the GAWDO QPSK shows better resilience under harsh conditions. It shows immunity to carrier offset drift and very few occurrences of phase ambiguity. The GAWDO QPSK demonstrates much better robustness to noise and adverse channel conditions. This is seen by its low bit errors which do not go beyond 8% regardless of the fading properties of the channel. This also implies that the GAWDO presents a system that has great coherence between the modulation and demodulation stages. Most traditional systems struggle with this aspect [32]. Data loss and phase misappropriations are also low when using the proposed GAWDO QPSK. Figure 4, Figure 5, Figure 6, Figure 7, Figure 8, Figure 9, Figure 10, Figure 11, Figure 12, Figure 13, Figure 14 and Figure 15 and Table 4 and Table 5 show that the enhanced modulation scheme gives more accurate results. With the increasing demand for bandwidth efficiency and higher data throughput [32,33], the proposed algorithm offers a solution that gives more accurate results regardless of the channel conditions and environment. The proposed methodology also offers solutions for systems that need the least possible amount of bit errors and distortions.

**Figure 16:**
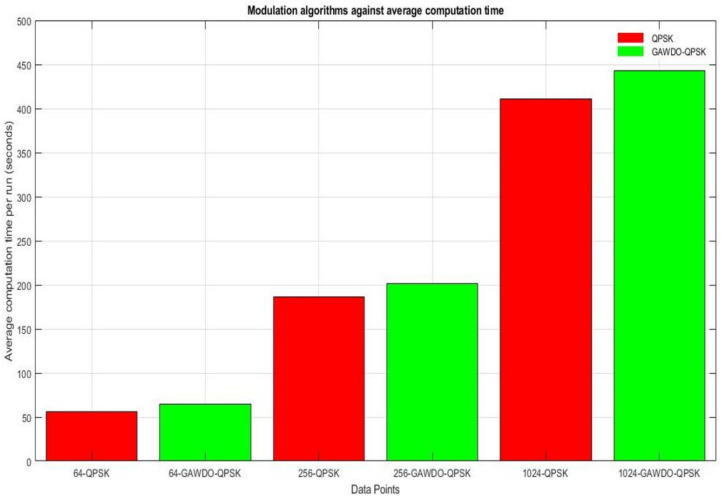
Modulation algorithms plotted against their average computational time.

During simulations, the GAWDO QPSK takes more computational time as compared to the customary QPSK. This is because the GAWDO undergoes numerous evolutionary cycles in determining the best phase assignments during modulation. However, for similar data points, the time difference between the GAWDO QPSK and the customary QPSK is less than 1 min as shown in Figure 16. This is not very significant when considering the quality of results that will be obtained.

## 6. Conclusions

This paper presented a methodology for deriving and assigning QPSK phases using a hybrid evolutionary algorithm. The wind-driven optimization was incorporated into the genetic algorithm as its selection function to help reduce the loss of good genetic material, mitigate stalling, and ensure good survival rates of the best individuals. A literature review of OFDM networks and the QPSK modulation technique showed that communication channels internally suffer from phase ambiguity and carrier offset drifts. These weaknesses result in a large BER because QPSK and most digital systems are based on binary data. The information distortions are further amplified by real-world occurrences, e.g., multipath fading, narrowband interferences, noise, and attenuation at high frequencies. The hybrid algorithm gave accurate results on the Ackley and Rastrigin benchmark functions and was subsequently used for optimum QPSK phase allocations. During data transmission, this enhanced QPSK produced results that were significantly better than the conventional QPSK. In a noiseless channel, the enhanced QPSK had 0% BER as compared to 7.8% for the conventional QPSK. In a Rayleigh fading channel with AWGN, the enhanced QPSK had a BER not exceeding 8% as compared to the conventional QPSK which gave BER magnitudes beyond 17%. The simulation results showed that the proposed modulation technique can easily overcome the challenges that the customary QPSK fails to subdue. In future work, we intend to use the GAWDO to enhance other higher-order modulation techniques, e.g., 256 QAM or 1024 QAM and investigate its performance over the existing methods. This is important because OFDM is being considered alongside orthogonal time-frequency spacing for the forthcoming radio technologies where there is a great need for efficiency in network connectivity and data transmission under high-Doppler scenarios.

## Figures and Tables

**Table 1:** Parameters of the genetic algorithm.

Parameter	Settings
Creation function	Uniform
Crossover function	Single point, 0.8
Population type	Double vector
Population size	1000
Pareto fraction	0.4
Selection function	WDO
Penalty factor	100
Migration fraction	0.2
Migration interval	20
Fitness scaling function	Top
Migration direction	Both
Mutation function	Uniform
Stall test	Geometric weighted
Stall time limit	60
Non-linear solver	Augmented Lagrangian

**Table 2:** Algorithm results on the Rastrigin function.

	Title 1	Title 2	Title 3	Title 4	Title 5
	(X_1_; X_2_)	(X_1_; X_2_)	(X_1_; X_2_)	(X_1_; X_2_)	(X_1_; X_2_)
GA	(0; 0.5)	(0; 0)	(−1; 3)	(0; 0)	(0; 0)
WDO	(0; 0)	(0; 1.5)	(0; 0)	(0; 0.06)	(0; 0)
GAWDO	(0; 0)	(0; 0.4)	(0; 0)	(0; 0)	(0; 0)

**Table 3:** Algorithm results on the Ackley function.

	Title 1	Title 2	Title 3	Title 4	Title 5
	(X_1_; X_2_)	(X_1_; X_2_)	(X_1_; X_2_)	(X_1_; X_2_)	(X_1_; X_2_)
GA	(0; 0)	(0; 0)	(0; 0)	(0; 0)	(0; 0)
WDO	(0; 0)	(0; 0)	(0; 0)	(0; 0)	(0; 0)
GAWDO	(0; 0)	(0; 0)	(0; 0)	(0; 0)	(0; 0)

**Table 4:** BER comparison between GAWDO and customary QPSK in a noiseless OFDM network.

Data Points	GAWDO QPSK % Error	Customary QPSK % Error
64	0	4.69
256	0	7.81
1024	0	4.2

**Table 5:** BER comparison between GAWDO and customary QPSK in a noisy OFDM network.

Data Points	GAWDO QPSK % Error	Customary QPSK % Error
64	1.56	9.38
256	3.51	17.58
1024	7.52	12.79
